# Understanding and overcoming the immunosuppressive effects of glioma induced immunosuppression

**DOI:** 10.1186/2051-1426-1-S1-P169

**Published:** 2013-11-07

**Authors:** Christopher Moertel, Junzhe Xi, Michael R Olin

**Affiliations:** 1 Pediatrics, University of Minnesota, Minneapolis, MN, USA

## 

Utilizing tumors as a source of vaccination antigens in immunotherapy has demonstrated promising results with minimal toxicity. Unfortunately, researchers have failed to overcome the overpowering suppressive effects of the tumor microenvironment. Recently we demonstrated that the proximity of the vaccination injection site to the primary tumor site dictates CD8 T-cell priming within the draining lymph node. Glioma-bearing mice vaccinated in tumor-distal sites had increased functional responses and survived significantly longer than glioma-bearing mice vaccinated in tumor-proximal sites due to immunosuppressive properties of the tumor inhibiting the priming of a tumoricidal response in the draining lymph nodes. Tumor induced immunosuppression is a complex interplay between the tumor and its surrounding microenvironment. We discovered an excess of soluble CD200 protein in the cerebral spinal fluid and cervical lymph nodes in tumor bearing mice. CD200 has been well characterized to be immunosuppressive in multiple graft rejection models, but has not been clearly defined as a factor in tumor-induced suppression. CD200 is highly expressed on a variety of human tumors including melanoma and glioblastoma and is co-expressed on tumor stem cells. CD200 is thought to be a mechanism by which these cells escape immune surveillance. Researchers have shown that specific peptide domains within the CD200 protein have antagonist activity (act as competitive inhibitors for CD200) for the CD200R. Together these data lead us to the development of our central hypothesis that tumor-induced immunosuppression is reversible using select competitive inhibitors of CD200. We have synthesized several competitive inhibitors by inoculating mice with tumor lysate vaccines. Preliminary data show that these inhibitor peptides derived from CD200 reverse immune suppression within lymph nodes, significantly extending survival of vaccinated glioma GL261-bearing and breast carcinoma EMT6-bearing mice. Due to the homology between human and mouse CD200, we further hypothesize that the use of CD200 inhibitors will be translatable to human cancers. IMPACT: This study is designed to elucidate and overcome the mechanism of tumor-induced immunosuppression and has major implications for the design of translational research approaches and future clinical trials. If our hypothesis is correct, the use of a competitive inhibitor may overcome the suppressive properties of the tumor in both the sentinel lymph nodes as well within the tumor microenvironment, ultimately leading to the development of novel therapeutics that increase the efficacy of cancer immunotherapy.

